# Design and Modeling of a Miniature Hydraulic Motor for Powering a Cutting Tool for Minimally Invasive Procedures

**DOI:** 10.3390/mi14071338

**Published:** 2023-06-29

**Authors:** Manjeera Vinnakota, Kishan Bellur, Sandra L. Starnes, Mark J. Schulz

**Affiliations:** 1 College of Engineering and Applied Sciences, University of Cincinnati, Cincinnati, OH 45221, USA; vinnakbm@mail.uc.edu (M.V.); schulzmk@ucmail.uc.edu (M.J.S.); 2 College of Medicine, University of Cincinnati, Cincinnati, OH 45221, USA; starnesl@ucmail.uc.edu

**Keywords:** miniature hydraulic motor, 3D printing, minimally invasive surgery, numerical modeling, ANSYS CFX, fluid structure interaction, immersed solid approach

## Abstract

Miniaturization of multifunctional instruments is key to evolving less invasive medical procedures. The current work outlines steps towards developing a miniature motor to power a cutting tool of a millimeter-scale robot/device (target outside diameter ~2 mm) for minimally invasive procedures. Multiple motor concepts were explored and ranked using a Pugh matrix. The single-rotor hydraulic design was deemed most viable for prototyping and scale-down to the target size. Prototypes were manufactured to be progressively smaller using additive manufacturing. The smallest prototype fabricated was 2:1 scale of the desired final size with a 2 mm outside diameter (OD) rotor and a device OD of 4 mm. The scaled prototypes with an 8 mm rotor were lab tested and achieved average speeds of 5000–6000 RPM at a flowrate of 15–18 mL/s and 45 PSI water pressure. Ansys CFX was used as a design tool to explore the parameter space and 3D transient simulations were implemented using the immersed solid method. The predicted rotor RPM from the modeling matched the experimental values within 3% error. The model was then used to develop performance curves for the miniature hydraulic motor. In summary, the single-rotor hydraulic design shows promise for miniaturization to the target 2 mm size.

## 1. Introduction

Traditional surgeries and diagnostic procedures are evolving to be less invasive to have improved patient outcomes [1,2]. Improving the reach and flexibility by developing miniature multifunctional laparoscopic instrumentation is an active research area [3,4]. Tetherless micro/nano devices are being developed for various hard-to-access applications [5,6]. Medical device companies are also innovating minimally invasive instrumentation and improving reach, flexibility and precision by introducing robotic surgical platforms such as Da Vinci [7], Versius [8], Ion [9] and Monarch [10]. Despite the advancements, drug delivery deep into a tumor is still an open problem. The tetherless micro/nano robots that travel through the blood stream have difficulty penetrating the tumor since it typically has less vascular density in central regions than the surrounding tissue [11].

In addition to overcoming the usual challenges of microdevices such as control, power etc. [12,13], biopsy with microdevices poses additional challenges since it requires mechanisms for tissue cutting and an ability to retrieve a sufficient sample for histology [14]. Unlike drug delivery, where microrobots can carry the drug to the tumor and circulate within the body until they are eliminated, a biopsy requires directed tissue cutting capability and a facility for carrying the collected sample until the device is retrieved along with the tissue specimen.

A miniature tethered robot, referred to as a millibot, a conceptual device of ~2 mm diameter with a 500 µm diameter cutter, that can traverse through tissue would provide direct access to target anatomical locations for the surgeons, be it for deep tumor penetration or biopsy. Such a device could create a path through the tissue to deploy micro/nano devices closer to the target. This approach of using a combination of tethered and untethered devices has been proposed in the literature [15]. The 2 mm target size for the device was a Voice of Customer from surgeons at the University of Cincinnati College of Medicine.

A millibot must possess several key functionalities such as tissue cutting, steering, navigation, drug delivery etc. Each of these individual design aspects is a research area with multiple open questions. The current paper outlines the efforts towards designing one of the subsystems i.e., a cutting module for a millibot. The cutting module consists of the hydraulic motor, fluid lines, and the cutter.

For traversing through tissue, a millibot requires a cutter (or cutters) to create a path for the device to be pushed forward. Three options for powering a cutter have been explored: (a) an external motor to power the cutter via a geared shaft or a flexible shaft (b) a miniature electric motor at the cutter and (c) a miniature hydraulic motor to power the cutter. In options (b) and (c) there is no transmission of power as the cutter is directly coupled with the motor distally.

There are medical applications such as atherectomy where catheter/guide wire-based devices utilize cutting tools in the 2.5 mm size range driven by flexible shafts [16,17]. However, these devices are designed to operate inside blood vessels.

In general, electrical motors are not suitable for miniaturizing. As the cross-sectional area of the conductor decreases, the resistance increases, and electrical energy input would be lost as heat. The smallest electric geared motor found had an outer diameter (OD) of 1.5 mm from Orbray Co., Ltd., but is no longer in production [18]. The next smallest is a 2.4 mm OD motor. Though this is in the target size range, there are unknowns regarding the interaction of motor heat with tissue and the motor’s biocompatibility. To overcome the challenges with waste heat, we propose a new design that uses a hydraulically powered cutting tip where the working fluid is either water or saline which are readily available in an operating room setting and are biocompatible.

The millibot cutting module explores hydraulic actuation of the micro cutter to achieve deep tissue penetration. Concepts generated for powering the cutter and the design journey of preliminary millibot cutting-tip prototypes are presented in this paper. It must be noted that the torque requirements would vary based on the type of tissue and cutter geometry. The initial design efforts were focused on miniaturization of a hydraulic motor and characterizing the available output torque and RPM. The miniature hydraulic motor was modeled in Ansys CFX to predict the performance in terms of rotational speed and torque for a given flow rate through the device. Results from the numerical analysis are also presented.

## 2. Materials and Methods

The objective of the preliminary design effort was to develop a miniature hydraulic motor that can operate with water or saline as a working fluid to power a cutting tool. Commercial hydraulic motors typically are installed in a fixed location and operate using standard hydraulic fluids, making them unsuitable for biocompatible applications. The size also is much larger than the ~2 mm target size for the final device. During the survey, the smallest gear motor found had a displacement of 0.5 cc/rev (~5 cm in length, product code: M1AAN0050FL20B01N, 0.25 cc/rev model is discontinued) from HPI (Chennevières-sur-Marne, Paris, France) [19].

The current work presents the first-generation designs of miniature motors operating with water, which can further be scaled down to the target size. These scaled-up prototypes enabled refinement of the design without the challenges of manufacturing and assembling micro components with every design iteration. 

**Figure 1:**
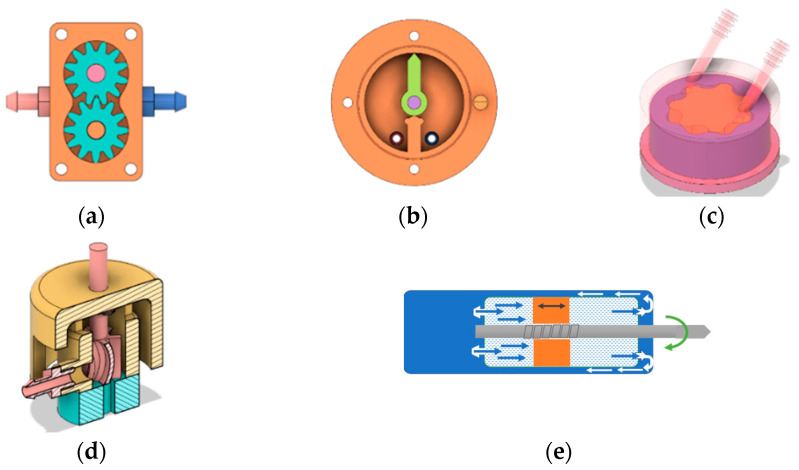
Hydraulic motor concepts for powering cutters: (**a**) External gear motor; (**b**) Vane motor; (**c**) Gerotor motor; (**d**) Single rotor; (**e**) Reciprocating element on a screw.

Multiple concepts for hydraulically powering the cutters were generated: external gear motor, vane motor, gerotor motor, single rotor (similar to a Pelton turbine) and reciprocating motor. Figure 1 shows the images of generated concepts. These designs were loosely based on the working principles of traditional hydraulic motor designs. A brief description for each of these concepts along with a comparison Pugh matrix and scoring methodology are discussed in Appendix A. The concepts were ranked to determine which ones were more viable for initial prototyping efforts. For prototyping and eventually scaling down to the target 2 mm size range, the single rotor design shown in Figure 1d, which received the highest score in the comparison matrix, was selected.

However, simple scaling down of traditional designs to the 2 mm target size range is not practical due to following reasons:

(1) The sealing elements cannot be implemented in the same way. The seals rely on compression which is achieved by tightening the fasteners. For a miniature device, there would not be any conventional fasteners in the assembly in order to reduce the overall size. Thus, the seals must be redesigned to work without compression or the motor design itself should be less sensitive to tight sealing.
(2) The part count must be reduced to simplify assembly. It is an extremely tedious task to assemble a 2 mm outside diameter device consisting of several parts.
(3) The impact of clearances inside the fluid chamber becomes significant on torque output.
(4) As length scale reduces, manufacturing tolerances become critical when dealing with interacting components.
(5) Secondary operations for increasing component precision are limited and add to the manufacturing cost for miniature components.

Scaling also poses design challenges such as maintaining component strength. Component strength is as much a design problem as it is a material science problem since the choices are limited to biocompatible materials available for micro manufacturing. 

Additive manufacturing was extensively used for fabricating the initial prototypes. Off-the-shelf standard parts were assembled with the components printed using Form 3+ (Formlabs, Somerville, MA, USA) printers to create preliminary cutting module prototypes. The prototypes were tested using a benchtop set-up. A Computational Fluid Dynamics (CFD) model was developed to aid in the design and development.

### 2.1. Single Rotor Design

**Figure 2:**
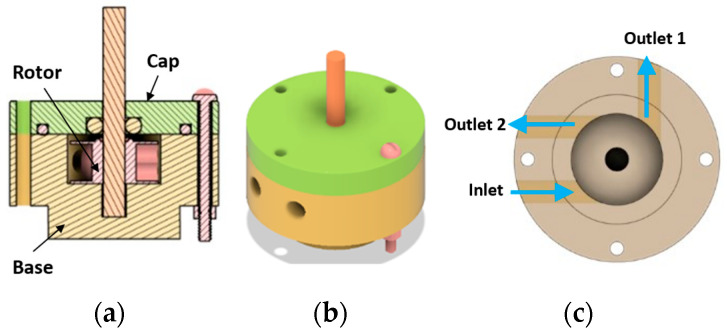
CAD of single rotor concept with radial outlets: (**a**) Cross section of the device showing rotor attached to cutting shaft, O-rings and fasteners; (**b**) Isometric view of the device, with drive shaft (no cutter) visible; (**c**) Base of the motor showing fluid lines.

The single rotor design is an impulse turbine like the Pelton wheel. The rotor has blades or cups that deflect the impinging water jet and convert the kinetic energy of the jet to the rotational energy of the rotor. The concepts consist of 3 main parts: Cap, Rotor and Base, as shown in Figure 2a, assembled either by fasteners or glue. Of all the hydraulic motor concepts considered, the single rotor design is the most forgiving in terms of manufacturing tolerances and defects. This is because the motor, being an impulse turbine, does not require tight sealing between cap and rotor to function. There is ample clearance in the design to accommodate the XY resolution (25 µm [20]) of a Form 3+ printer that was used for prototyping. This eliminates the need for secondary operations in addition to support removal for closely fitting the components. However, manual removal of the supports leaves remnants on the components increasing internal friction of the device. Hence, supportless printing would be utilized for scaling down.

**Figure 3:**
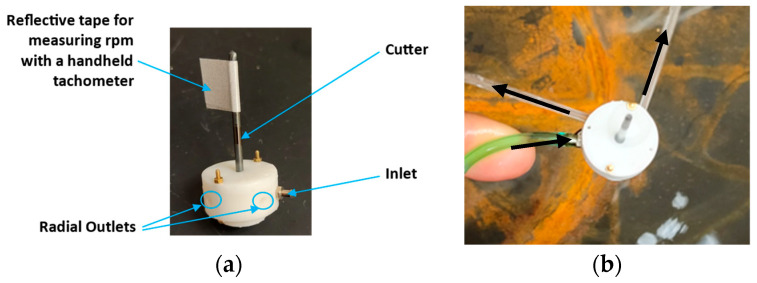
Fabricated prototype: (**a**) Miniature hydraulic motor (8 mm rotor, OD 19 mm) fabricated using 3D printed parts along with off-the-shelf parts; (**b**) Water inlet and outlet through the device.

Two versions of this design were modeled and prototyped, one with radial outlets (Figure 2) and the other with inline outlets. The radial outlets version was simpler to manufacture and assemble as it used small bolts and nuts for assembly. This version had an outside diameter (OD) of 19 mm. Standard O-rings were used for reducing leakage of working fluid from inside the device. A standard drill bit of 2 mm diameter was press fitted into the 3D printed rotor as a representative cutter. Standard white resin (LGPWH04, Formlabs, Somerville, MA, USA) was used for printing these parts. Fluid flow lines are shown in blue in Figure 2c. Even though the outside diameter of the prototype was 19 mm, the rotor diameter was only 8 mm (Figure 3). The rest of the land was for accommodating the fasteners. To put it in perspective, a typical rotor of a dental handpiece air turbine is 8 to 12 mm in diameter [21]. In this design, two outlets were provided for reducing the back flow and resistance to fluid exiting the device. The inline outlets concept had a more compact profile since glue (Gorilla super glue no drip gel) was used instead of fasteners to reduce the number of components.

**Figure 4:**
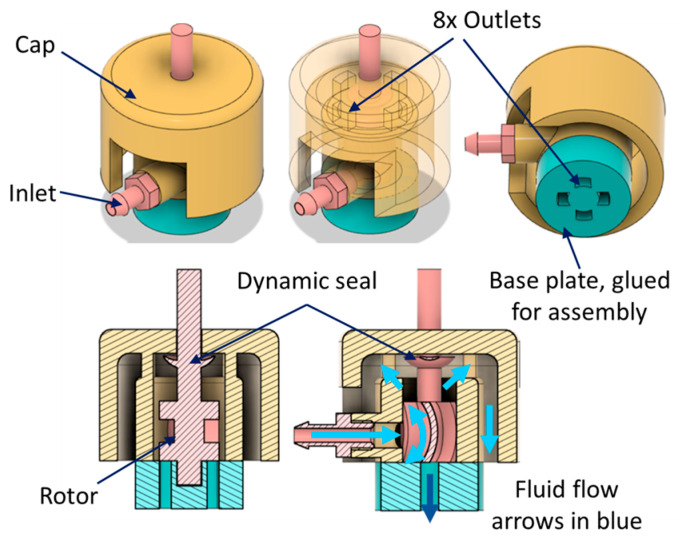
CAD of single rotor design with inline outlets.

**Figure 5:**
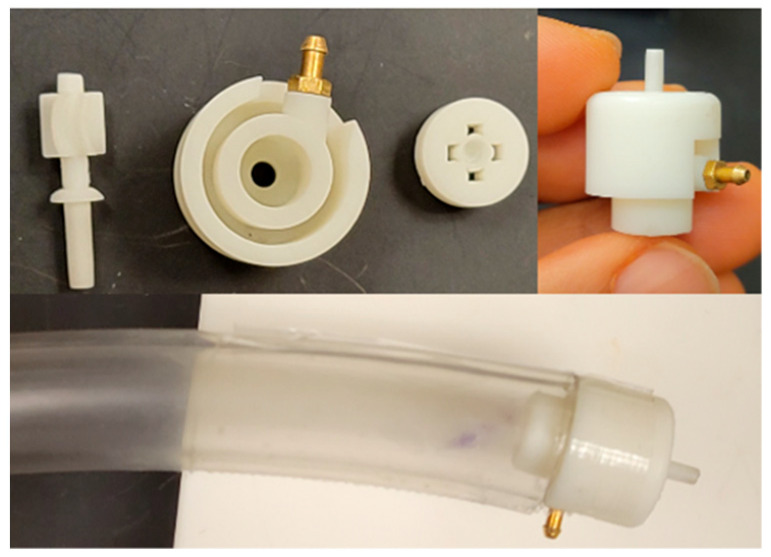
3D printed components and assembled device (OD 15 mm) attached to a tether to be more representative of the final device with inline outlets.

The inline outlets design (Figure 4) used a dynamic seal instead of O-rings. The seal was integrated with the rotor and was of the same material. No assembly was required as it was printed along with the rotor. As the rotor rotates, it lifts up and the seal ‘cups’ the cutter hole in the cap, thus cutting off the leakage path. This device had an OD of 15 mm. Figure 5 shows the 3D printed parts in standard white resin (LGPWH04) and the assembled prototype with a tether attached. 

**Figure 6:**
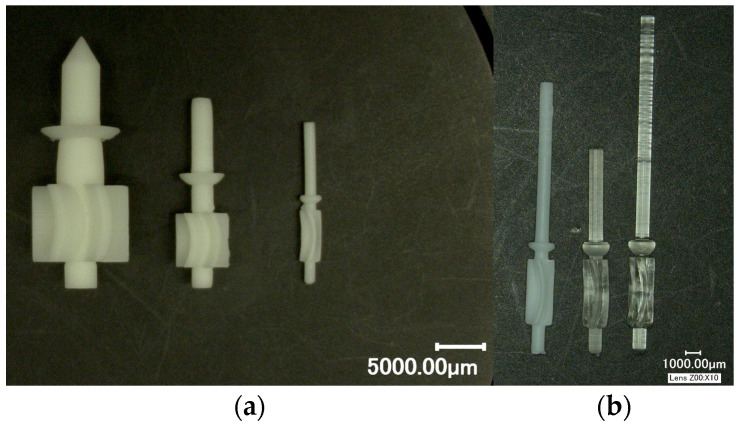
3D printed rotors: (**a**) Progressively smaller rotors for scaling down; OD of the largest rotor was 10 mm, and the smallest was 2 mm; (**b**) 2 mm OD rotors printed in 3 different materials.

**Figure 7:**
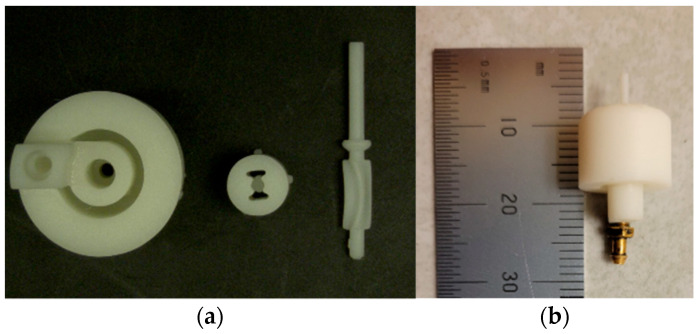
OD 11 mm device with inline inlet and outlets: (**a**) 3D printed parts, 2 mm rotor; (**b**) Assembled prototype.

In an effort to scale down the design, a 2 mm OD rotor was printed. The rotor was 3D printed using 3 different Formlabs materials (Formlabs, Somerville, MA, USA): (1) standard white (LGPWH04) (2) biocompatible resin (FLBMAM01) and (3) high temperature resin (FLHTAM02). There were no discernable differences in the printed rotors of the 3 materials in terms of feature resolution. The rotor had 5 cups of 500 µm width and 5 mm in length. Diameter of the cutter shaft was 1 mm. Prototype parts are shown below in Figure 6 and Figure 7. The device in Figure 7 had an OD of 11 mm, even though the rotor was only 2 mm, as it still utilized an off-the-shelf barb fitting for connecting the water line.

**Figure 8:**
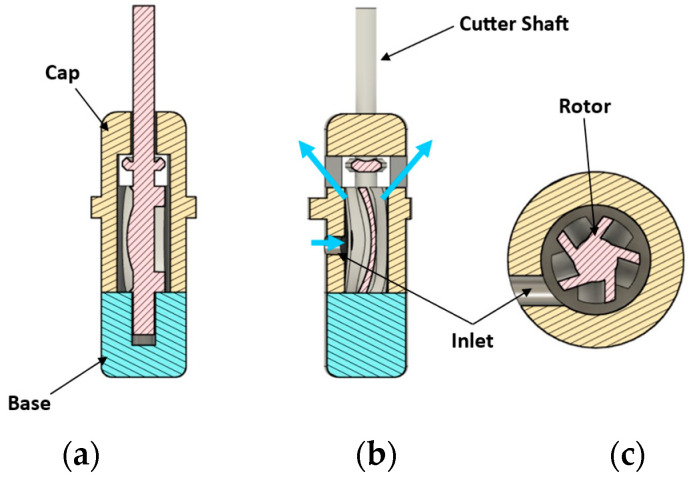
CAD of 4 mm OD single rotor design with 2 mm OD rotor: (**a**) Cross section showing rotor, cap, and base; (**b**) Fluid flow lines in the plane of inlet; (**c**) Section through inlet of 850 µm diameter. Blue arrows indicate the flow path.

The device diameter was further reduced from 11 mm to 4 mm in the next concept shown in Figure 8, where water exits the device instead of being routed internally and discharged via the tube. This concept had a nominal diameter of 4 mm at the tip. The cap had 4 outlets (of which only two are seen in Figure 8) through which the water could exit the device once the work had been extracted by the rotor. Flow direction is indicated by blue arrows.

**Figure 9:**
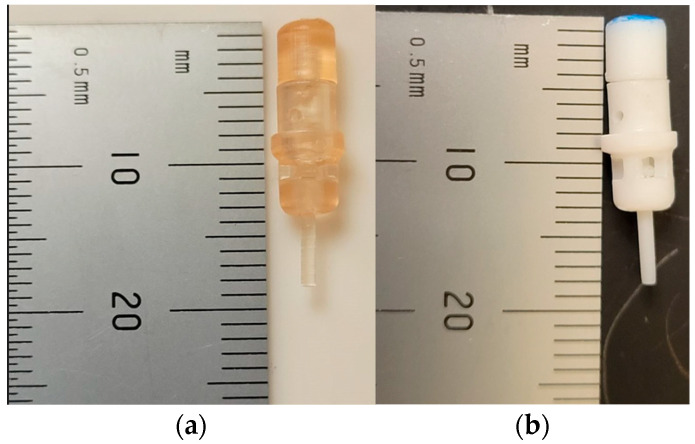
OD 4 mm device with 2 mm rotor: (**a**) Printed in biomedical resin; (**b**) Standard white resin.

The manufactured prototype is shown in Figure 9. All the components were printed in both biocompatible resin and standard white resins.

### 2.2. Experimental Set-Up 

**Figure 10:**
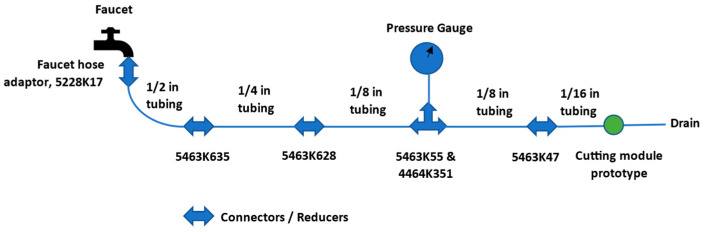
Experimental set-up schematic with McMaster-Carr part numbers for reducers.

For experimentation with prototypes, a test set-up was constructed using standard off-the-shelf components. Flexible silicone tubing was used with various pipe fittings and connectors to attach prototypes to the building water supply. The set-up schematic including the McMaster-Carr part numbers is shown in Figure 10. The pressure was continuously monitored using a pressure gauge (part number 3847K71, 0–200 PSI, McMaster-Carr, Elmhurst, IL, USA) located before the inlet of the hydraulic motor prototype. When the faucet was turned on and water flow started, the prototype converted the kinetic energy of the flow into mechanical energy as evidenced by rotation in the cutter shaft and water exited the motor through the 2 outlets. The entire set-up was placed inside a sink to drain the water coming out of the device. Flow rate was controlled by opening and closing of the faucet. When the faucet was in the fully open position, attainable peak pressure was 75 ± 5 PSI. The motor inlet pressure, flow rate and cutter RPM were recorded during the experiments.

**Figure 11:**
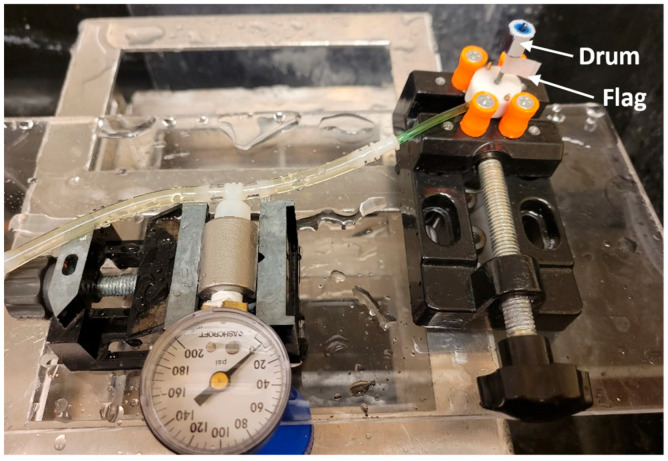
Experimental bench top set-up.

The RPM was measured by a handheld tachometer (model number: LH900RF, EHDIS, Amazon, Seattle, WA, USA). A reflective tape was attached to the cutter to form a flag of 1 cm × 1 cm for pointing the tachometer laser. A 3D printed drum of 9 mm diameter and 8.75 mm height blackened using a sharpie, with a 5 mm wide reflective tape along the length, was also attached to the shaft to take concurrent readings of RPM both using the reflective flag and drum. This set-up is shown in Figure 11. 

The assembled device was connected to the 1/16 in ID tube of 1.3 in length. A typical test procedure first began with turning on the faucet to the desired flow rate and pressure. As the cutting module prototype started to rotate, maximum RPM value reported by the handheld tachometer was recorded. The flow through the motor was calculated by collecting water from the outlets for either a 5 or 10 s time period. A small amount of leakage was observed at the cutter due to the lack of precise fit between the O-ring and cutter. When the flow started through the device, it was observed that a drop formed around the cutter shaft and spread into a layer on most of the cap’s top surface before dripping to the sides. It took 5–10 s for a layer to form on top when the pressure was set to 45 ± 5 PSI. Assuming the height of water layer to be 1 mm, a leak rate of 0.06 mL/s was estimated. The data collected is presented in results. It must be noted that the RPM, pressure, and flow rate readings were not concurrently taken. The pressure was recorded first and then the RPM. Typically, the flow rate was measured at the beginning and end of the experiment.

### 2.3. Computational Fluid Dynamics (CFD) Model

While the experiments enable preliminary testing, computational models could be leveraged to speed up prototyping and subsequent testing. Here, ANSYS CFX (ANSYS, Canonsburg, PA, USA), a computational fluid dynamics (CFD) tool specifically developed for turbomachinery [22], is used to model the cutting module performance. CFX has successfully been used to model gear pumps by Yoon, Y. et al. [23]. Nishi, Y. et al. [24] modeled the performance of a dental air turbine using ANSYS CFX. Juraeva, M. et al. [25,26] implemented a DOE (Design of Experiments) to optimize the dental air turbine designs. The goal of creating a CFD model was to be able to predict the rotor RPM and power output for a given set of design parameters including but not limited to geometry (diameter/size), hydraulic (mass flowrate, pressure, etc.) and fluid properties (viscosity, density, etc.). A model for the radial outlets design was first created using the experimental data as initial inputs. Once validated, the CFD model could then be adapted to other scenarios and even used to validate the scaled-down versions of the device. 

CFD models were created for the radial outlet single rotor designs with 8 mm rotor in ANSYS CFX version 2022 R2. These were 3-Dimensional turbulent models involving fluid structure interaction. Immersed solid methodology was used to model the interaction between rotor and fluid. The immersed solids in CFX help to model rigid bodies that move through the fluid domain. The immersed solid occupies the same volume of space as part of the fluid domain and the mesh of immersed solid overlaps that of the fluid domain. The motion of the solid body (rotor) is influenced by two types of forces: (1) shear stress from the fluid surrounding it and (2) external torques, or forces imposed on the solid. Initially, the solid body is set to be stationary. As the transient simulation progresses, the position of the immersed solid is updated as governed by the rigid body equations. At each time step, the new position is updated in the fluid domain by identifying nodes that fall inside the solid body to apply immersed solid source terms to map the fluid velocity and the solid velocity. This enables a 2-way coupling between the overlapping solid and fluid domains. Detail on the immersed body method is available in the ANSYS CFX solver modeling guide [27]. Here, the rotor was modeled as an immersed solid that could rotate about the device axis (all other degrees of freedom were constrained) in the fluid domain. No slip boundary conditions were used at all walls. The fluid velocity was set to 0 m/s at a relative pressure of 0 PSI for initial conditions and the model was solved until a steady-state angular velocity of rotor was observed. 

The CFX model incorporated resistance or load on the motor as an opposing external torque on the rotor to analyze how the free RPM of cutting shaft changes w.r.t applied torque. The k-ϵ turbulence model was used in conjunction with rigid body solver to obtain a solution. The Simo Wong algorithm [28] was used for the angular momentum equations with a coupling control of every coefficient loop. Z translation of the rotor was constrained. The momentum source scaling factor was set to 10. The residual target was set to 10^−5^. An unstructured mesh with linear elements was used. Other simulation parameters are in Table 1. For the mesh independence study, the external torque was set to 0.0001 Nm. The number of elements varied from 226,755 to 926,661 elements, refinement by a factor of approximately 4. The mesh independence study is presented in the CFX results Section 3.2. The analysis results were compared with the experimental data in terms of rotor RPM and flow rates through outlets 1 and 2 (Figure 2c) and are described in the proceeding sections.

## 3. Results and Discussion

### 3.1. Experimental Data

The radial outlets prototype motor was connected to the test set-up and RPM and flowrate data was collected at different pressures. The accuracy of the pressure gauge, as reported by the manufacturer, is ±2 percent. The accuracy of the tachometer is ±0.02 percent when measuring at a distance from 2–20 inches (50–500 mm). Time was recorded using a stopwatch with a least count of 0.1 s. The least count of the graduated measuring cylinder used for collecting fluid exiting the motor is 2 mL. Flow rate was calculated as the volume of fluid exiting through both the outlets divided by the time for which it was collected. The data collected in Table 2 was recorded with only the reflective flag attached to the output shaft for RPM measurement using the handheld tachometer.

It was observed that the pressure fluctuated ±5 PSI when set at a particular value. The “Max” value reported using a handheld tachometer was recorded during experiments.

**Figure 12:**
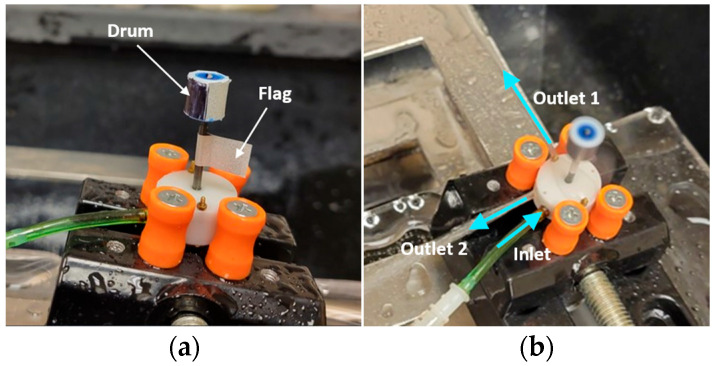
Experimental set-up for RPM measurement: (**a**) Drum and flag arrangement; (**b**) Inlet and outlets indicated by blue arrows.

The data in Table 3 was recorded with both drum and tape attached to the output shaft of the radial outlets prototype for RPM measurement as shown in Figure 12. No significant difference was observed between the two types of measurement. Fluctuations in flowrate were observed even if the pressure was set at a certain value. If the pressure drifted during the experiment, it was adjusted back to the set value before continuing with the data collection.

**Figure 13:**
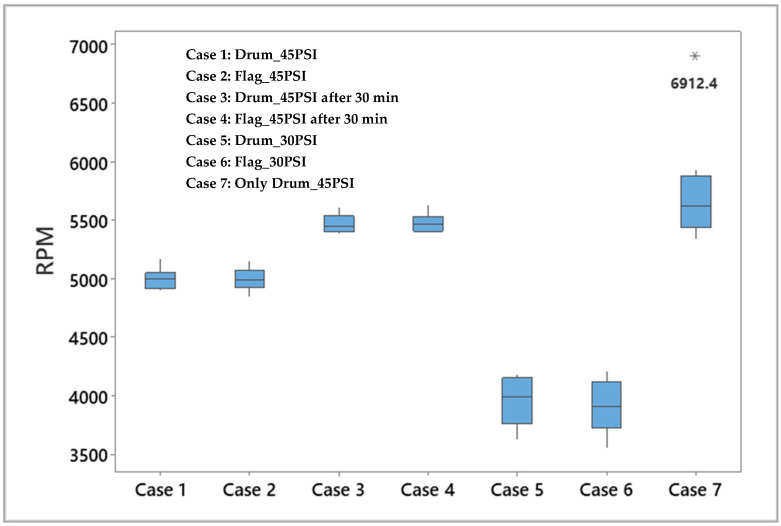
Box plots of experimental data summarized in Table 3. * Indicates outlier.

When the prototype was allowed to completely dry between tests, scales formed inside because of tap water drying out. This increased the frictional resistance of the device. Fluctuation in RPM was less when the motor was running for some time. In Figure 13, “Only Drum_45PSI” corresponds to the data collected when the prototype was tested at time (t) = 0 min, “Drum_45PSI” and “Flag_45PSI” correspond to data collected after 15 min of running the prototype and “Drum_45PSI_30 min” and “Flag_45PSI_30 min” correspond to data collected after running the prototype for 30 min. When the pressure was set at 45 PSI, an upward drift in RPM was noticed as the experiment progressed as can be seen in the first 4 box plots of Figure 13. This might be due to smoothing of rough spots reducing the internal friction. The spread in RPM readings was higher at lower flow rates (box plots corresponding to Drum_30PSI, Flag_30PSI). The 30 PSI data was collected after the experiment with the prototype at 45 PSI. The data (Supplementary material: Spreadsheet S3) was analyzed using Minitab statistical software (version 20.4) for generating box plots in Figure 13 and descriptive statistics in Table 4. 

### 3.2. ANSYS CFX Results

The mesh independence study was carried out by refining the mesh by a factor of 4.1, increasing the number of elements from 226,755 to 926,661 elements. The relative error in rotor steady-state angular velocity between a mesh with 726,138 elements and the finest mesh with 926,661 elements is 2.06%. The change in absolute value of average rotor steady state angular velocity between these 2 grids is 11.48 rad/s (109.63 RPM). It must be noted that the spread in RPM from the experiments was 225–300 (Table 4) for a flow rate between 15–18 mL/s at 45 ± 5 PSI which was much higher than the modeling uncertainty of 109.63 RPM.

**Figure 14:**
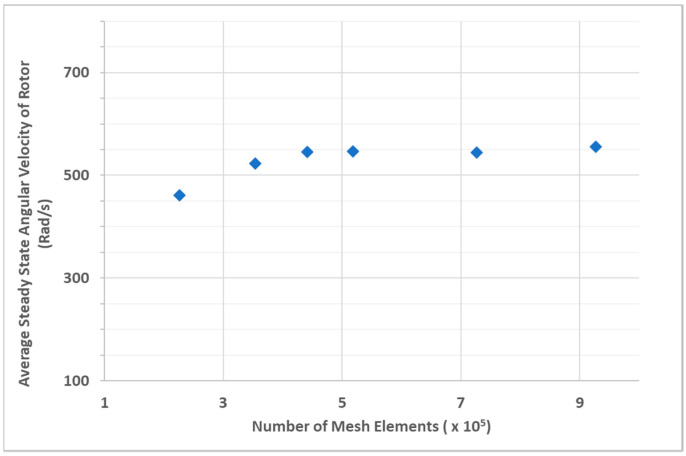
Average steady-state angular velocity of rotor w.r.t Number of mesh elements.

Figure 14 shows the change in steady-state rotor angular velocity w.r.t number of mesh elements. For subsequent analyses, the grid size of 726,138 elements was used. 

**Figure 15:**
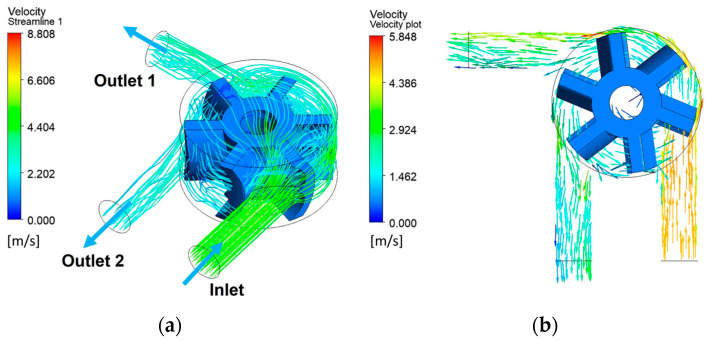
Simulation results: (**a**) 3D streamlines inside the device; (**b**) Velocity vectors.

For a specified inlet flowrate and pressure outlet, the fluid-structure interaction simulation was set up such that the angular velocity of the rotor was an output. Figure 15a shows the simulated 3D streamlines in the device as the rotor rotates against an external torque of 0.0001 Nm and Figure 15b shows the velocity vectors on a plane at mid height. Supplementary Video S1 animates rotor movement corresponding to the flow that develops through the device. Supplementary Video S2 shows the rotor rotation in the prototype during experimentation.

**Figure 16:**
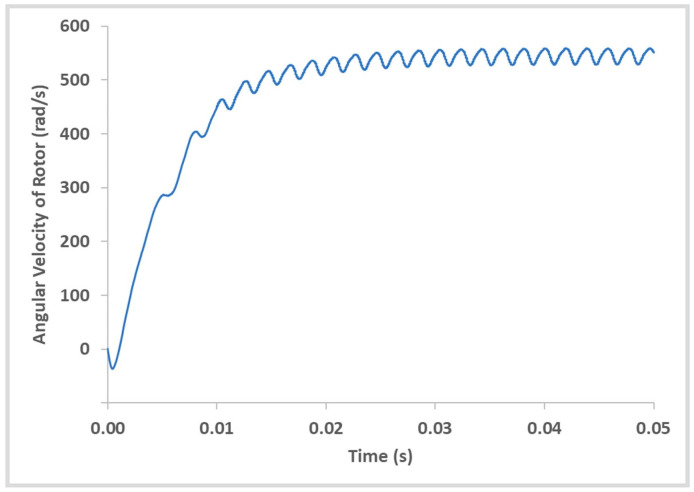
Rotor Angular Velocity (rad/s) vs. Time (s).

As the blade rotates, the jet is no longer perpendicular to it. This causes a momentary drop in the angular velocity but is eventually restored when the next blade passes through. This results in an oscillatory pattern (Figure 16). The periodicity of the oscillation matches with the angular separation of blades. i.e., the peaks correspond to the jet striking perpendicular to the blades on the rotor. A typical transient response shown in Figure 16 is with an opposing external torque of 0.0001 Nm applied on the rotor.

The maximum rotor angular velocity for a flow rate of 16.8 mL/s was 5336.96 RPM from the simulation. The corresponding experimentally measured value was 5472.8 (Table 3) with a standard deviation of 80.8 RPM (Table 4). Hence, the model is in good agreement with experimental results both qualitatively and quantitatively, at least to within experimental uncertainty.

Uncertainty in flowrate measurements (δq) was calculated using rules for error propagation (Equation (1)). Since the uncertainty in time measurement (δt) was independent of the uncertainty in flow measurement (δf):

(1) $$\frac{\delta q}{q}=\sqrt{\;{\left(\frac{\delta f}{f}\right)}^{2}+{\left(\frac{\delta t}{t}\right)}^{2}}$$

δf is the least count of the measuring cylinder, 2 mL. δt is the least count of the stopwatch used, 0.1 s. Therefore, uncertainty in mass flowrate is ±0.0002 kg/s. The relative error in mass flowrate between the mesh with 726,138 elements and the finest mesh with 926,661 elements was calculated to be 1.5% at outlet 1 and 2.3% at outlet 2. The relative error in rotor steady-state angular velocity between the mesh with 726,138 elements and the finest mesh with 926,661 elements was calculated to be 2.06%. Therefore, a 2% discretization error was considered for calculating the error bars on predicted mass flowrate and RPM values from the analysis in Table 5. The standard deviation calculated from the experimental data takes into account the random variability and measurement error. From Table 4, the standard deviation is 80.8 RPM. Therefore, ±3 standard deviations (±242 RPM) would account for 99.7% of the experimental data, assuming data is normally distributed. 

It must be noted that there was slight leakage at the cutter shaft O-ring that approximated to 0.06 mL/s (i.e., 0.00006 kg/s) based on calculations. This leakage would reduce the amount of fluid exiting through the outlets by a small amount and might explain the higher percentage error at outlet 1 compared to the analysis where no leakage was modeled. Error was calculated using the nominal values w.r.t the analysis values since the numerical model represents an ideal scenario without leaks.

**Figure 17:**
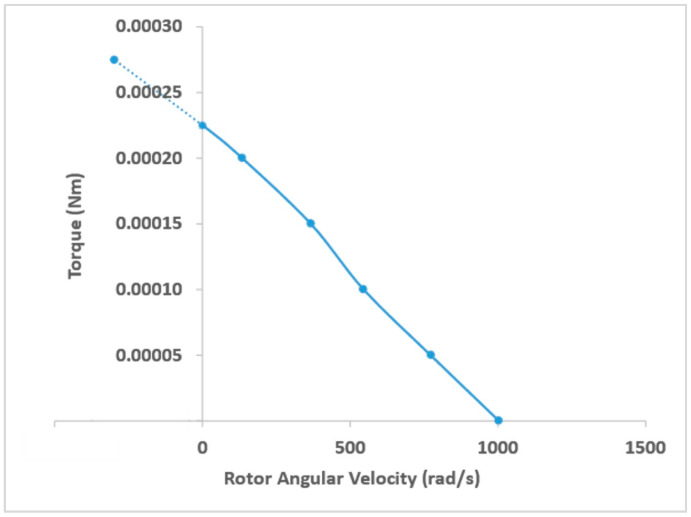
Torque (Nm) vs. Rotor Angular Velocity (rad/s).

Performance curves were generated for the miniature motor using the CFX models. Figure 17 shows Torque (Nm) vs. Angular Velocity (rad/s). The stall torque was predicted to be 0.000225 Nm. 

**Figure 18:**
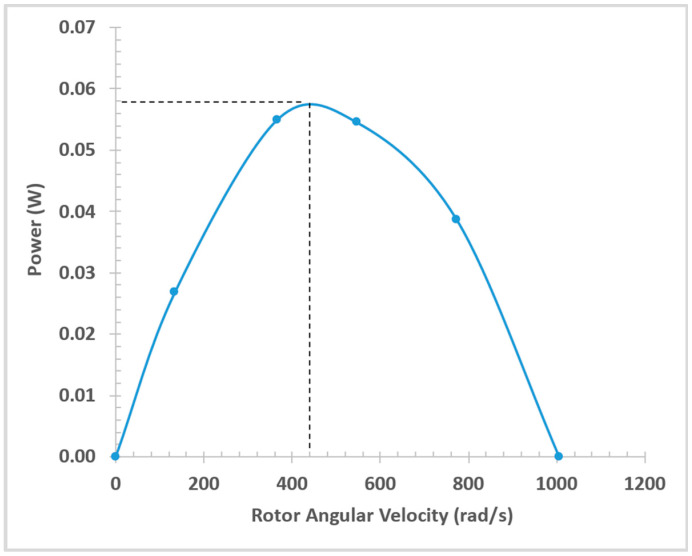
Power (W) vs. Rotor Angular Velocity (rad/s).

Figure 18 shows the Power (W) vs. Rotor Angular Velocity (rad/s). The peak power output from the cutting module was found to be 0.058 W at 440 rad/s (4200 RPM). The graph was generated using 6 data points from simulations and the points were connected with smooth lines only as a visual aid based on the expected trend. 

## 4. Conclusions

For miniaturizing the hydraulic motors to power a cutting tool, multiple motor concepts were generated and narrowed down to the single rotor design because of its simplicity in function and design. It was selected based on concept comparison using a Pugh matrix (Appendix A) and was deemed most suitable for scaling to the target size of 2 mm. The first generation of single rotor prototypes were 19 mm in OD with an 8 mm rotor attached to a 2 mm output shaft. Standard off-the-shelf parts such as O-rings and drill bits were used in combination with the 3D printed components to manufacture the 19 mm OD prototypes. These prototypes were tested using a benchtop set-up and could rotate at 5000–6000 RPM at a flowrate of 15–18 mL/s and 45 PSI water pressure. As the design evolved towards the target size, the OD of the hydraulic motor was reduced from 19 mm to 15 mm to 11 mm to 4 mm. The rotor dimensions reduced from 8 mm to 5 mm to 2 mm. The 4 mm OD prototype is 2:1 the final size with all 3D printed custom components. These miniature motors can also be run with compressed air. No data has been collected yet with air as the working fluid.

The 19 mm OD design was extensively tested at different pressures and flowrates using water as working fluid. The data was used to validate an ANSYS CFX model that was developed. The CFX model was utilized to characterize the miniature hydraulic motor and the peak power output from the cutting module was found to be 0.058 W at 4200 RPM. The free angular velocity of rotor in an ideal frictionless condition was predicted to be 9596 RPM and stall torque was found to be 0.000225 Nm. 

Future work involves experimentation with the fabricated prototypes of 2:1 (4 mm OD) design using the same benchtop set-up and developing CFX models for this design. The design would then be scaled to the final size of ~2 mm and the prototypes would be fabricated. CFX models would play a critical role in characterizing ~2 mm micromotor designs as experimentation and measurement would become very challenging due to the smaller magnitudes of various parameters of interest. Another key aspect is to evaluate the cutting-module torque output w.r.t the tissue cutting forces. The cutting forces would greatly depend on the nature of tissue and cutter geometry.

Overall, the single rotor hydraulic motor shows promise for scaling down to a 2 mm size range to power a 500 µm cutting tool for use in minimally invasive procedures. 

## 5. Patents

Invention disclosure is being processed though the University of Cincinnati Office of Innovation (Reference number: UC 2023–121, June 2023).

## Figures and Tables

**Table 1:** CFX simulation parameters.

Variable	Ansys CFX
Total Time	0.05 s
Time Step	5 μs (adjusted to keep RMS Courant Number < 1)
Inlet Boundary	Mass flow 0.0168 kg/s (based on experiments)
Outlet Boundary	Opening, 1 atm
Element order	Linear

**Table 2:** Experimental data collected at different pressures and flowrates.

Pressure (PSI)	Outlet 1 Flow (mL) *	Outlet 2 Flow (mL) *	RPM	Total Flowrate Outlets 1, 2 (mL/s)
75	58	56	7484.9	22.8
	58	58	7908.8	23.2
	60	58	7365.0	23.6
50	52	38	5236.4	18.0
	48	46	5773.2	18.8
	50	46	5507.9	19.2
40	46	38	4356.3	16.8
	48	36	4415.2	16.8
	46	38	4488.7	16.8
30	38	34	2772.0	14.4
	40	32	2738.9	14.4
	40	32	2491.8	14.4

* Measured for 5 s.

**Table 3:** Experimental measurements with drum and tape.

Pressure (PSI)	Average of Max RPM with Drum	Average of Max RPM with Flag	Average Total Flowrate, Outlets 1, 2 (mL/s)
45 *	5758.7	N/A	17.6
45	4997.4	4993.5	17.1
45 **	5472.8	5479.4	15.7
30	3947.4	3908.6	13.7

* Only drum attached to the shaft; ** After running motor for 30 min.

**Table 4:** Descriptive statistics for the experimental data.

Variable	Sample Size	Mean	SE Mean *	St Dev **	Minimum	Maximum	Range
RPM Drum_45PSI	9	4997.37	29.5	88.6	4896.7	5175.0	278.3
RPM Flag_45PSI	9	4993.52	31.4	94.3	4846.5	5151.0	304.5
RPM Drum_45PSI_after 30 min	12	5472.77	23.3	80.8	5386.0	5611.0	225.0
RPM Flag_45PSI_after 30 min	12	5479.38	22.5	78.1	5389.4	5632.0	242.6
RPM Drum_30 PSI	14	3947.39	53.0	198.4	3628.0	4176.3	548.3
RPM Flag_30 PSI	14	3908.56	58.6	219.2	3552.0	4202.4	650.4
RPM Only Drum_45PSI	9	5758.72	159.1	477.3	5342.4	6912.4	1570.0

* Standard error in mean; ** Standard deviation.

**Table 5:** Experiment vs. Analysis results.

Variable	Ansys CFX Analysis	Experiment	% Error
Massflow outlet 1 (kg/s)	0.01014 ± 0.00020	0.00895 ± 0.00020	11.7%
Massflow outlet 2 (kg/s)	0.00666 ± 0.00013	0.00675 ± 0.00020	1.4%
Maximum RPM	5336.96 ± 107	5472.77 * ± 242	2.5%

* Average value of the “Max” readings recorded.

**Figure A1:**
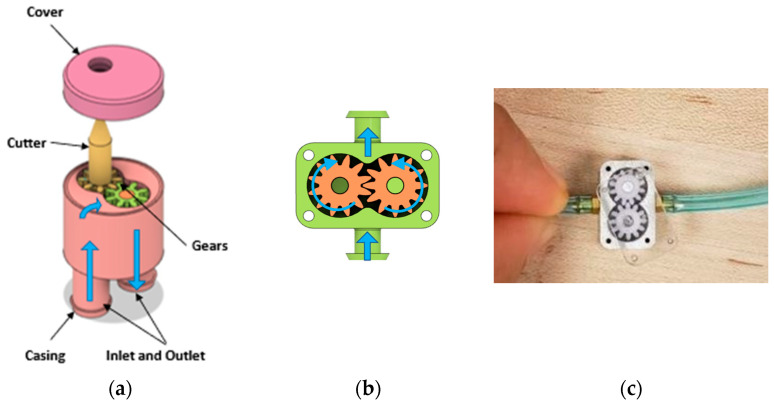
Gear motor concept: (**a**) CAD of a gear motor with circular outer profile with blue flow arrows; (**b**) CAD design of a gear motor with rectangular outer profile showing theoretical gear rotation; (**c**) Manufactured prototype.

**Figure A2:**
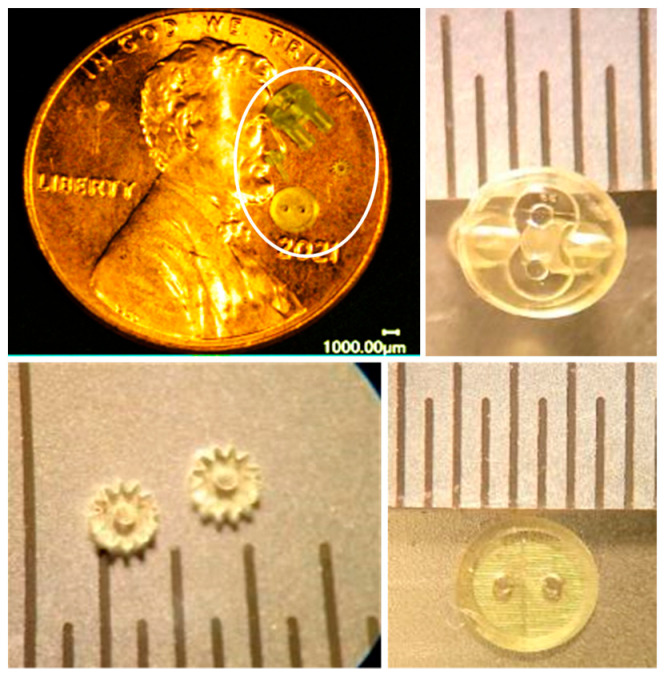
2.5 mm OD gear motor components manufactured using µ3D Printing.

**Figure A3:**
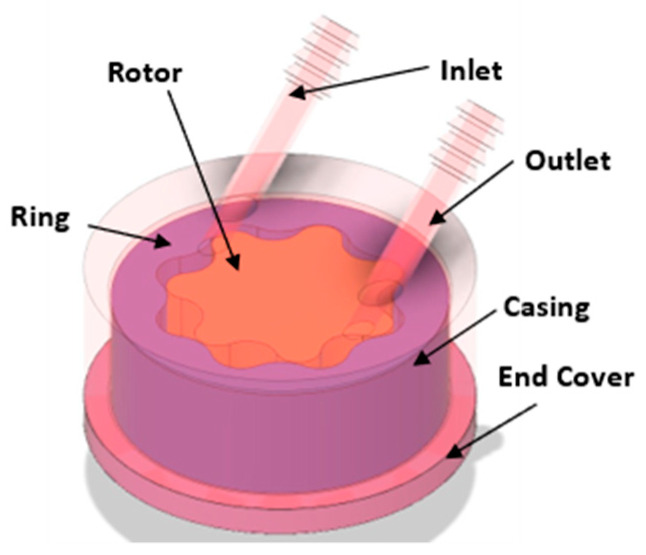
CAD of gerotor motor concept.

**Figure A4:**
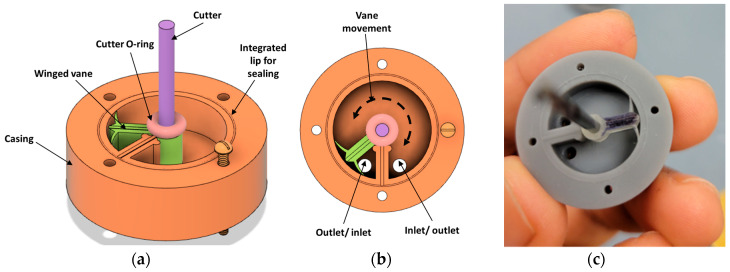
Vane motor: (**a**) CAD of the concept; (**b**) Vane movement indicated by the dashed arrow as the inlet and outlet are swapped; (**c**) 3D printed and assembled prototype.

**Figure A5:**
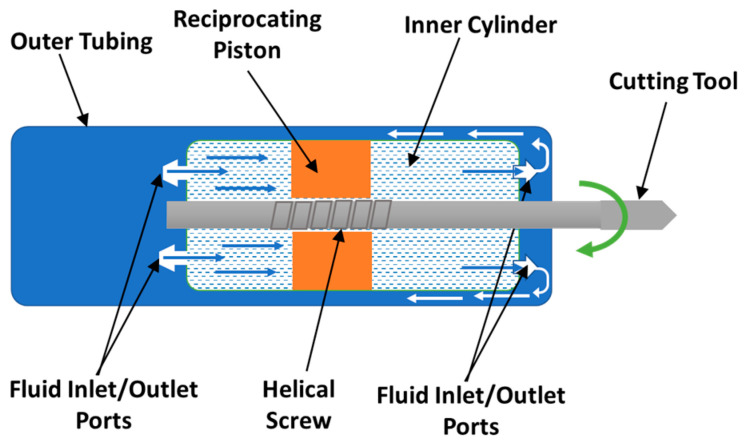
Reciprocating element motor concept.

**Figure A6:**
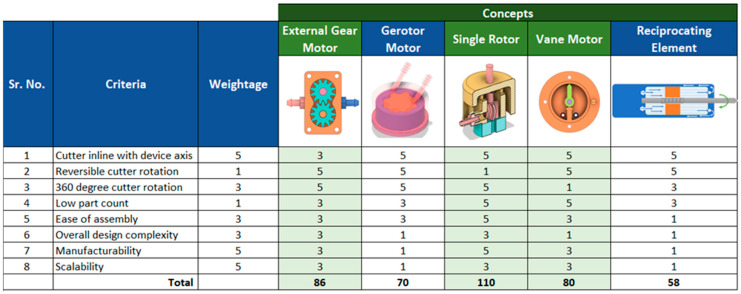
Concept comparison Pugh matrix.

## Data Availability

The data presented in this study are available on request from the corresponding author. All the data are not publicly available due to invention disclosure being processed.

## Supplementary Materials

The following supporting information can be downloaded at: https://www.mdpi.com/article/10.3390/mi14071338/s1, Video S1: 3D Streamlines; Video S2: Experiment; Spreadsheet S3: Data for Figure 13 and Table 4.

## Appendix A

A brief description of the generated concepts is presented here.

Gear Motor: The working principle of an external gear motor is simple and well established. The concept generated consisted of 2 meshed spur gears enclosed in a casing. The inlet and outlet were opposed to each other, similar to the traditional layout (Figure A1). A standard 2 mm drill bit was press-fitted as the cutter to one of the gears and rotated along a parallel off-center axis to the device. The casing was machined in aluminum and standard barbs were used for attaching the inlet and outlet to water lines. The top cover was attached using 4 bolts and nuts. The overall dimensions were 16 mm × 10 mm. The outer profile was rectangular for the ease of machining internally. It is only cosmetic and does not affect the device function. During the experimentation, the direction of rotation was opposite to the theoretical rotation. The root cause was found to be large internal clearances causing fluid to leak around the gears. Manufacturing tolerances make it difficult to maintain the tight clearances that are critical to the design. As the parts become smaller, the gear tooth width to radial gap at tooth tip ratio becomes very sensitive to the tolerances. A 2.5 mm OD version of the gear motor prototype was manufactured using µ3D Printing (Figure A2). However, this could not be assembled due to interference between the cutter shaft and cover.

Gerotor Motor: This concept is based on the traditional gerotor motor that is also well established. It consisted of a rotor and a ring that are eccentric to each other forming fluid pockets of variable size along the circumference (Figure A3). The pressure differential in these pockets drives the motor. The components need to be of very high precision to maintain the fit and rolling motion. The type of finish and precision would require multiple secondary operations for the components.

Single Rotor Motor: The single rotor design is based on the working principle of an impulse turbine like the Pelton wheel. The kinetic energy of the water jet is converted to the rotational energy of the rotor by means of blades or cups. The design mainly consisted of a rotor, cap and base that were attached either using fasteners or glue. This concept was selected for initial prototyping and was presented in detail in the main body of this research paper.

Vane Motor: This concept consisted of an oscillating vane attached to the cutter (Figure A4). The inlet and outlet were perpendicular to the plane of vane rotation. The wings on the vane provided a dynamic seal to minimize internal leakage from high pressure to low pressure regions. The vane rotated until it came in contact with the stop and then the inlet/outlet were reversed for the vane to rotate in the opposite direction. This was a problem during testing as the frequency of oscillation depended on the speed of inlet/outlet switching. In addition, if there is backpressure from the outlet region, the torque output from the device goes down. To maintain the low pressure on the outlet side and high pressure on the inlet side with an ability to rapidly switch requires a sophisticated hydraulic circuit. Even though the concept is attractive as the rotation can be reversed and has fewer components than the gear motor, the complexity in the hydraulic circuit is a barrier to its realization.

Reciprocating Element Motor: This concept consisted of a piston mounted on a helical screw (Figure A5). As the fluid pushes the piston it rotates the helical screw which in turn rotates the cutter. When the inlet and outlet are reversed, the piston moves in the opposite direction, thus reversing the rotation of the helical screw. This concept has the same problem as that of the vane motor, requiring a complex hydraulic circuit to switch the inlet and outlet at a high rate to reverse the cutter rotation.

The goal of this Pugh matrix (Figure A6) exercise was to prioritize the prototyping efforts that will yield designs with higher desirable characteristics and success rate. The generated concepts were pitted against each other using 8 criteria such as type of cutter rotation, manufacturability, scalability etc. Weights of 1, 3, 5 were assigned to the criteria with 5 being most critical and 1 being least critical for functioning of the device.

A score of 1, 3, 5 (low, medium, high) was assigned for evaluating a particular concept w.r.t a given criteria. For instance, the external gear motor has an off-center cutter axis, so a medium score of 3 was assigned to criterion 1 where having a cutter along the device axis is considered desirable. Similarly, for the criterion ‘Manufacturability’ a medium score of 3 was assigned as the design is sensitive to dimensional tolerances and poses manufacturing challenges. The component tolerances can negatively impact the amount of clearance in the device and therefore the internal leakages.

The vane motor concept is also sensitive to tolerances. Hence, this concept was also assigned a medium score of 3 for manufacturability. It also requires a hydraulic circuit for suction on the outlet when the inlet is open and vice versa for reversing the cutter rotation. This increases the overall complexity of the system and it was assigned a low score of 1 for the ‘Overall design complexity’ criterion.

The single rotor design received a low score of 1 for the ‘Reversible cutter rotation’ criterion as the rotor design does not facilitate reverse rotation.

For the scaled-up versions, concepts with the top 3 scores, i.e., the external gear motor, single rotor and the vane motor designs were chosen for prototyping. The gerotor and reciprocating element motors were not prototyped. For scaling down to the target 2 mm size range, the single rotor design which received the highest score was selected.
